# Patient-reported outcomes in a Pituitary Tumor Center of Excellence (PTCOE)–aligned pituitary clinic compared with general endocrinology care

**DOI:** 10.1007/s11102-026-01692-8

**Published:** 2026-05-23

**Authors:** Ali Nail Yagci, Aslihan Pekmezci, Meliha Melin Uygur, Sebnem Burhan, Melda Celik, Fatma Aktas Yagci, Mazhar Muslum Tuna, Mutlu Niyazoglu, Esra Hatipoglu

**Affiliations:** 1 Department of Internal Medicine, University of Health Sciences, Basaksehir Cam and Sakura City Hospital, Istanbul, Turkey; 2 Department of Endocrinology and Metabolism, University of Health Sciences, Basaksehir Cam and Sakura City Hospital, Istanbul, Turkey; 3 Department of Endocrinology and Metabolism Diseases, School of Medicine, Recep Tayyip Erdoğan University, Rize, Turkey; 4 Department of Endocrinology and Metabolism, University of Health Sciences, Umraniye Training and Research Hospital, Istanbul, Turkey; 5 University of Health Sciences, Pituitary Disorders Research Center, Istanbul, Turkey

**Keywords:** Illness perception, Outpatient satisfaction, Patient-reported outcomes, Pituitary adenoma, PTCOE, Quality of life

## Abstract

**Purpose:**

Pituitary Tumor Centers of Excellence (PTCOE) were developed to standardize multidisciplinary care for pituitary disorders; however, their impact on patient-reported outcomes remains insufficiently characterized. This study aimed to compare health-related quality of life, illness perception, and outpatient satisfaction between patients followed in a PTCOE-aligned, though not formally accredited, specialized pituitary clinic and those managed in general endocrinology clinics, and to identify organizational factors associated with these outcomes.

**Methods:**

In this cross-sectional study, 175 adults with prolactinoma (*n* = 70), non-functioning pituitary adenoma (*n* = 54), acromegaly (*n* = 35) and Cushing’s disease (*n* = 16) were evaluated across two tertiary endocrinology centers. Patient-reported outcomes were assessed using validated Turkish versions of the SF-36, Illness Perception Scale, and Outpatient Satisfaction Scale.

**Results:**

Certain baseline clinical profiles differed significantly between the two groups, with patients in the specialized clinic exhibiting higher rates of pituitary surgery (63.9% vs. 32.1%, *p* = 0.001) and hypopituitarism (28.9% vs. 14.1%, *p* = 0.02). Patients in the specialized clinic showed significantly lower SF-36 Role Physical, Role Emotional, Social Functioning, and Bodily Pain scores (all *p* ≤ 0.03). Illness-perception patterns also diverged, with higher Timeline, Consequences, and Treatment Control scores in the specialized clinic. Outpatient satisfaction, however, was consistently higher across all subscales in the specialized clinic (all *p* ≤ 0.006). In multivariable analyses, clinic type, sex, hypopituitarism, surgical history, and follow-up duration independently contributed to variation in multiple patient-reported outcome domains.

**Conclusion:**

PTCOE-aligned specialized care was associated with more favorable illness representations and higher patient satisfaction, even among cases with greater disease burden. This underscores the need for integrating patient-reported outcomes into the evaluation and refinement of pituitary care models.

## Introduction

 Pituitary disorders comprise a heterogeneous group of conditions arising from functional or structural abnormalities of the pituitary gland, a central regulator of endocrine homeostasis [1]. Their clinical spectrum encompasses hormonally active and non-functioning pituitary tumors as well as rarer inflammatory or cystic lesions, each presenting distinct diagnostic and therapeutic challenges [2, 3]. These conditions often follow an insidious course and require lifelong follow-up, with possible hormonal deficiencies, mass effects, and systemic comorbidities contributing to substantial physical, psychological, and social burden [4, 5]. Despite advances in medical and surgical care, many patients continue to report persistent symptoms and impaired quality of life, underscoring the chronic and multidimensional nature of the pituitary diseases [6].

Given their complexity and rarity, pituitary disorders necessitate coordinated, highly specialized management; however, access to such expertise can varies considerably across healthcare systems [3]. To address this variability, the Pituitary Tumor Centers of Excellence (PTCOE) model was introduced by the Pituitary Society in 2017 as a structured framework aimed at centralizing expertise and standardizing clinical pathways [7]. PTCOEs integrate endocrinology, neurosurgery, neuroradiology, neuropathology, ophthalmology, and radiation oncology to improve diagnostic accuracy, streamline management decisions, and enhance patient safety [8]. Building on this model, Giustina et al. recently translated the initial qualitative descriptions into quantifiable benchmarks, including defined staffing requirements, minimum surgical volumes, and acceptable complication thresholds, providing a measurable foundation for future accreditation efforts [3, 9, 10]. Subsequent work has extended the PTCOE concept to disease-specific management: dedicated frameworks for acromegaly and Cushing’s disease care within PTCOEs have been proposed, outlining standards for medical therapy, multidisciplinary decision-making, and patient monitoring [11–13]. Collectively, these contributions have established PTCOEs not only as structural entities but as operational models with defined clinical standards and measurable outcomes.

However, these structural and operational benchmarks primarily capture institutional performance and do not fully reflect the patient’s lived experience. Existing evaluations of PTCOE effectiveness have largely emphasized biochemical remission, tumor control, and postoperative morbidity, with limited attention to patient-reported outcomes (PROs) such as satisfaction with care or health-related quality of life [6, 8, 14, 15]. This represents a clinically relevant gap, as many patients continue to experience treatment burden, persistent symptoms, and psychosocial challenges even after biochemical control is achieved [16, 17]. Traditional biomedical indicators therefore offer an incomplete picture of care quality in pituitary disease.

A deeper understanding of patient perspectives is essential for assessing the true impact of specialized pituitary care. Patient-reported outcome measures capture domains, such as communication quality, care coordination, treatment convenience, and emotional well-being, that cannot be evaluated through clinical metrics alone [18, 19]. Integrating these measures into PTCOE assessments is crucial for validating the model, informing accreditation standards, and guiding improvements in patient-centered care. Yet, to date, no comparative studies have systematically examined whether PTCOE-aligned clinics provide superior patient-reported satisfaction or health-related quality of life relative to general endocrinology clinics, marking a clear gap in the literature.

Therefore, this study aims to determine whether specialized pituitary care, delivered in a clinic aligned with PTCOE principles, is associated with measurable advantages in patient-reported satisfaction and quality of life compared with general endocrinology care, and to identify organizational or clinical factors that may underlie any observed differences.

## Materials and methods

### Study design and setting

This cross-sectional comparative study was conducted between November 2023 and April 2024 at two tertiary endocrinology centers in Istanbul, Turkey: Basaksehir Cam and Sakura City Hospital (BCSCH) and Umraniye Training and Research Hospital (UTRH). The study was designed to compare patient-reported satisfaction and health-related quality of life between a specialized pituitary clinic operating within a PTCOE-aligned model at BCSCH and standard general endocrinology clinics at UTRH. Patients at BCSCH were referred primarily through neurosurgery, internal medicine, or direct outpatient endocrinology referrals. Following pituitary surgery or initial diagnosis, patients with complex or surgically managed disease were retained for ongoing multidisciplinary follow-up within the specialized pituitary program. At UTRH, patients were referred from primary care or self-referred, and follow-up was conducted within the standard outpatient endocrinology service without a dedicated pituitary subspecialty pathway. No systematic cross-referral between the two centers occurred during the study period.

### Study population

Adult patients (≥ 18 years) with a confirmed diagnosis of prolactinoma, non-functioning pituitary adenoma (NFPA), acromegaly, or Cushing’s disease who were under routine follow-up at either center were eligible. Inclusion required a minimum outpatient follow-up duration of 6 months in the respective clinic. Diagnoses were established using standard clinical, biochemical, and radiological criteria. In non-operated NFPA cases, diagnosis was established based on typical pituitary MRI features consistent with adenoma and the absence of biochemical hypersecretion or other sellar pathology [20, 21]. Patients were assigned to one of two groups according to their routine follow-up setting:

1. the specialized pituitary clinic group, representing care delivered within a structured multidisciplinary model at BCSCH. BCSCH functions as a dedicated pituitary-focused center operating in alignment with core PTCOE principles. The institution provides (i) subspecialized pituitary clinics staffed by endocrinologists with focused expertise, (ii) a dedicated high-volume pituitary neurosurgery program with coordinated surgical–endocrine management pathways, (iii) formal multidisciplinary evaluation involving core disciplines (endocrinology, neurosurgery, neuroradiology, and neuropathology) supported by ophthalmology and specialized endocrine nursing services, (iv) standardized diagnostic and longitudinal follow-up protocols for pituitary tumors, and (v) systematic monitoring of long-term clinical outcomes and patient-reported measures. Collectively, these features position BCSCH as a center operating within the framework consistent with internationally recognized PTCOE models.
2. the general endocrinology clinic group, representing standard outpatient endocrine care at UTRH.

Exclusion criteria included: (1) pituitary or sellar pathology other than the four adenoma subtypes; (2) severe cognitive or psychiatric impairment precluding questionnaire completion; (3) acute pituitary apoplexy or other emergent conditions at assessment; (4) inability to comprehend or complete questionnaires; and (5) incomplete primary outcome data.

### Study protocol and data collection

Clinical and demographic variables were extracted from electronic medical records, including age, sex, disease duration, remission status, hypopituitarism, prior pituitary surgery or radiotherapy, and current treatments. Hypopituitarism was defined according to established biochemical evidence of anterior pituitary hormone deficiency and/or the documented need for corresponding hormone replacement therapy [22]. Hormonal profiles appropriate to each adenoma subtype were recorded in accordance with guideline-based recommendations, using the most recent anterior pituitary panel obtained within three months of questionnaire administration.

Disease remission and activity status were defined according to current guideline-based biochemical and clinical criteria for each adenoma subtype. For prolactinoma, remission required the absence of clinical symptoms, ≥ 50% tumor reduction on follow-up imaging, and normal serum prolactin (< 20 ng/mL) [23, 24]. For NFPA, remission was evaluated only in surgically treated patients and defined as the absence of residual or recurrent tumor [20, 21]. In acromegaly, postoperative biochemical remission required age-adjusted normalization of insulin-like growth factor-1 (IGF-1) at 12 weeks and, when available, adequate growth hormone (GH) suppression during a 75-g oral glucose tolerance test (OGTT) (nadir GH < 0.4 µg/L for body mass index (BMI) < 25 kg/m² and < 0.2 µg/L for BMI ≥ 25 kg/m²). Patients who did not achieve remission at the 3-month postoperative evaluation were commenced on medical therapy, and resistance was defined as the failure to normalize IGF-1 levels and/or the persistence of random GH levels above 1 µg/L despite the use of appropriate, standard medical treatment [25–27]. For Cushing’s disease, remission was defined as serum cortisol < 1.8 µg/dL following a 1-mg overnight dexamethasone suppression test, at least two midnight salivary cortisol measurements < 0.4 µg/dL, and a 24-hour urinary free cortisol < 45 µg/24 h; failure of any criterion indicated persistent disease [28].

### Patient-reported outcome measures

Three validated patient-reported outcome measures were administered to assess health-related quality of life, illness perception, and outpatient treatment satisfaction. All instruments were used in their Turkish validated versions, each with established psychometric reliability and content validity for use in clinical research.

Health-related quality of life was measured using the 36-Item Short Form Health Survey (SF-36), a widely utilized multidimensional instrument originally developed within the Medical Outcomes Study [29]. The scale evaluates eight domains, including physical functioning, role limitations due to physical or emotional problems, vitality, mental health, social functioning, bodily pain, and general health perception [29]. Domain scores were transformed to a 0–100 scale, with higher scores reflecting better perceived health status [29]. The Turkish adaptation of the SF-36 has demonstrated strong reliability and validity, supporting its use in both population-based and disease-specific clinical studies [30].

Illness perception was assessed using the Illness Perception Scale (IPS), originally developed by Weinman et al. and later refined by Moss-Morris et al. [31, 32]. The IPS captures three major components of patients’ cognitive and emotional representations of illness: (1) symptom identity, (2) illness representation subscales, including timeline, consequences, personal control, treatment control, coherence, cyclical patterns, and emotional response, and (3) causal-attribution subscales [32]. Higher scores on relevant subscales indicate more negative illness perceptions [32]. The Turkish version has demonstrated robust psychometric validity and reliability, supporting its use among patients with chronic endocrine disorders [33].

Patient satisfaction with outpatient care was evaluated using the Outpatient Satisfaction Scale, a 29-item instrument comprising five subscales: appointment procedures, effectiveness of the clinical encounter, staff attitude, waiting time, and overall satisfaction [34]. Items are rated on a five-point Likert scale, with higher scores indicating greater satisfaction [34]. The Turkish adaptation has demonstrated high internal consistency and is widely applied in ambulatory care research [34].

In addition to the standardized questionnaires, patients followed in the specialized pituitary clinic were asked to respond to a single supplementary Likert-type item evaluating the perceived contribution of support staff to the quality of clinical follow-up.

### Statistical analysis

Statistical analyses were performed using SPSS Statistics for Windows, Version 27.0 (IBM Corp., Armonk, NY, USA). Normality was assessed using the Kolmogorov–Smirnov test. Normally distributed data were presented as mean ± standard deviation and compared using Student’s t-test. Non-normally distributed variables were expressed as median [interquartile range], and compared using the Mann–Whitney U test or Kruskal–Wallis test, with post-hoc pairwise Mann–Whitney U testing when appropriate. Associations between continuous variables were evaluated using Spearman’s rank correlation. Categorical variables were compared using the Pearson chi-square test or Fisher’s exact test as appropriate. Multivariable linear regression analyses were conducted to evaluate independent predictors of patient-reported outcomes. A two-sided p-value < 0.05 was considered statistically significant.

### Ethical approval

The study was conducted in accordance with the ethical standards of the 1975 Declaration of Helsinki and was approved by the Ethics Committee of Basaksehir Cam and Sakura City Hospital (Approval No: E96317027-514.10-225914733; Date: 3 October 2023). Written informed consent was obtained from all participants prior to questionnaire administration and data collection.

## Results

A total of 175 patients were included in the final analysis, of whom 97 were followed in the specialized pituitary clinic at BCSCH and 78 were managed in general endocrinology clinics at UTRH. The diagnostic distribution comprised 70 patients with prolactinoma (31 BCSCH, 39 UTRH), 54 with NFPA (32 BCSCH, 22 UTRH), 35 with acromegaly (24 BCSCH, 11 UTRH), and 16 with Cushing’s disease (10 BCSCH, 6 UTRH) (*p* = 0.09).

### Demographic and clinical characteristics of the study population

A detailed summary of demographic and clinical characteristics is presented in Table 1. The mean age was comparable between the groups (*p* = 0.7). Sex distribution differed significantly, with a higher proportion of female patients in the general clinic group (79.5% vs. 62.9%; *p* = 0.02). The median duration of outpatient follow-up was significantly longer in the general clinics compared with the specialized pituitary clinic (*p* = 0.03), while disease duration did not differ significantly between the groups (*p* = 0.3). The prevalence of comorbid conditions was higher in the specialized pituitary clinic than in general endocrinology clinics (60.8% vs. 47.4%), without reaching statistical significance (*p* = 0.08).

**Table 1:** Demographic and Clinical Characteristics of the Study Population

Variable	Specialized Pituitary Clinic at BCSCH (*n*=97)	General Endocrinology Clinics at URTH (*n*=78)	*p*-value
**Gender**, female/male, n (%)	61 (62.9) / 36 (37.1)	62 (79.5) / 16 (20.5)	0.02*
**Age** (years)^a^	42.43 ± 12.32	41.68 ± 13.45	0.7
**Educational level**, n (%) No formal education Primary school Middle school High school University Graduate education	4 (4.1) 37 (38.1) 11 (11.3) 29 (29.9) 13 (13.4) 3 (3.1)	2 (2.6) 24 (30.8) 9 (11.5) 19 (24.4) 21 (26.9) 3 (3.8)	0.3
**Pituitary adenoma subtype**, n (%) Prolactinoma Acromegaly Cushing’s disease Non-functioning pituitary adenoma	31 (32) 24 (24.7) 10 (10.3) 32 (33)	39 (50) 11 (14.1) 6 (7.7) 22 (28.2)	0.09
**Disease duration** (months)^a^	48 [24-84]	43 [17-84]	0.3
**Presence of comorbid conditions**, n (%)	59 (60.8)	37 (47.4)	0.08
**Remission status**^**b**^, n (%) Remission Persistent disease	52 (53.6) 32 (33)	39 (50) 25 (32.1)	0.7
**Presence of pituitary surgery**, n (%)	62 (63.9)	25 (32.1)	0.001*
**Presence of radiotherapy (Gamma Knife /CyberKnife)**, n (%)	3 (3.1)	3 (3.8)	0.8
**Current medical therapy**^**c**^, n (%) Cabergoline Octreotide Lanreotide Pasireotide Pegvisomant No current medical therapy	27 (27.8) 3 (3.1) 11 (11.3) 1 (1) 3 (3.1) 57 (58.8)	32 (41) 4 (5.1) 0 (0) 0 (0) 1 (1.3) 41 (52.6)	0.07 0.5 0.002* 0.4 0.4 0.4
**Presence of hypopituitarism**, n (%)	28 (28.9)	11 (14.1)	0.02*
**Hypopituitarism subtypes**, n (%) Diabetes insipidus Growth hormone deficiency Central hypothyroidism Central adrenal insufficiency Hypogonadotropic hypogonadism	0 (0) 0 (0) 12 (12.4) 19 (19.6) 9 (9.3)	3 (3.8) 0 (0) 10 (12.8) 8 (10.3) 6 (7.7)	0.05 - 0.9 0.09 0.7
**Outpatient follow-up durations** (months)^a^	17 [13-27]	23.5 [11.75-58.5]	0.03*

^a^ Results are presented as median and interquartile range [IQR]^b^ In patients with non-functioning pituitary adenomas, remission status was evaluated only in those who underwent surgical treatment. Accordingly, remission assessment was not applicable in 13 patients (13.4%) from BCSCH and 14 patients (17.9%) from UTRH^c^ Patients may have received more than one medical therapy* *p* < 0.05 was considered statistically significant. Abbreviations: BCSCH, Basaksehir Cam and Sakura City Hospital; UTRH, Umraniye Training and Research Hospital

Patients in both groups had similar remission rates (*p* = 0.7). Pituitary operations were carried out with greater frequency in patients attending the specialized clinic (*p* = 0.001). Hypopituitarism was also more prevalent among patients in the specialized clinic group (*p* = 0.02).

### Comparison of SF-36 quality of life domain scores between centers

When centers were compared, patients receiving care at the specialized pituitary clinic displayed notably lower scores in four SF-36 domains: Role Physical, Role Emotional, Social Functioning, and Bodily Pain (*p* = 0.001, *p* = 0.004, *p* = 0.03, and *p* = 0.03, respectively) (Table 2).

**Table 2:** Comparison of SF-36 domains, illness perception, and outpatient satisfaction subscale scores between centers

Variable	Specialized Pituitary Clinic at BCSCH (*n*=97)	General Endocrinology Clinics at URTH (*n*=78)	*p*-value
**SF-36 Domains** Physical Functioning Role Physical Role Emotional Vitality Mental Health Social Functioning Bodily Pain General Health Perception	85 [65-95] 25 [0-100] 33.3 [0-100] 40 [25-65] 56 [42-76] 62.5 [37.5-100] 57.5 [35-90] 50 [35-65]	85 [68.75-100] 100 [43.75-100] 83.33 [33.3-100] 52.5 [30-65] 60 [44-73] 75 [50-100] 67.5 [52.5-92.5] 60 [35-70]	0.1 0.001* 0.004* 0.4 0.6 0.03* 0.03* 0.05
**Illness Perception Subscales** Symptom Identity Timeline (acute/chronic) Consequences Personal Control Treatment Control Coherence Cyclical Patterns Emotional Response Psychological Attributions Risk Factors Immunity Chance	6 [2-8] 3.83 [2-4.58] 3.16 [2.5-3.83] 3.5 [2.91-4] 4.3 [4-4.8] 3.6 [2.6-4] 3.25 [2.12-4] 3 [2.33-3.83] 2.33 [1.5-3.5] 1.57 [1.35-2.07] 2 [1.33-2.67] 1.5 [1-2.5]	4 [1-7.25] 2.33 [2-3.83] 2.5 [2.12-3.33] 3.67 [3.16-4] 4 [4-4.2] 3.2 [2-4] 2.87 [2.25-3.5] 3 [2-3.71] 2.67 [2.33-3.33] 2.07 [1.85-2.28] 2 [2-2.67] 2 [1.5-2.5]	0.2 0.001* < 0.001* 0.16 < 0.001* 0.11 0.33 0.65 0.07 < 0.001* 0.167 0.036
**Outpatient Satisfaction Subscales** Appointment Procedures Effective Clinical Encounter Staff Attitude Waiting Time and Counseling Overall Satisfaction Total Outpatient Satisfaction Scale Score	5 [3.4-5] 5 [4.72-5] 4.67 [4-5] 3.67 [3-4] 4.6 [4-5] 4.58 [4.22-4.8]	3.2 [2.8-4] 4 [3.98-4.66] 4 [4-4.3] 3.3 [2.67-3.33] 3.6 [3-4] 3.72 [3.51-4.11]	< 0.001* < 0.001* 0.002* 0.006* < 0.001* < 0.001*

All results are presented as median and interquartile range [IQR]* *p* < 0.05 was considered statistically significant. Abbreviations: BCSCH, Basaksehir Cam and Sakura City Hospital; UTRH, Umraniye Training and Research Hospital

To explore whether variables that differed significantly between the two groups, namely sex, history of pituitary surgery, presence of hypopituitarism, duration of outpatient follow-up, and type of clinic, were associated with these domain scores, all variables were entered into multivariable regression models. In the adjusted analyses, Role Physical scores were lower among female patients, those with hypopituitarism, and patients followed in the specialized pituitary clinic (*p* = 0.01, *p* = 0.02, and *p* = 0.001, respectively). Role Emotional scores were likewise reduced among female patients, those with hypopituitarism, and patients receiving care in the specialized clinic (*p* = 0.01, *p* = 0.03, and *p* = 0.001, respectively). For Social Functioning, lower scores were independently associated with female sex, hypopituitarism, and follow-up in the specialized clinic (*p* = 0.002, *p* = 0.02, and *p* = 0.01, respectively). The scores on the Bodily Pain domain were lower among female patients and those followed in the specialized clinic (*p* < 0.001, and *p* = 0.01, respectively). Comprehensive regression coefficients and 95% confidence intervals are presented in Table 3.

**Table 3:** Multivariable regression analyses of factors associated with SF-36 domain scores

**Role Physical** *Model summary*: R² = 0.13; ANOVA *p* < 0.001* **Predictor**	β	95% CI	*p*-value
Sex (female) History of pituitary surgery Presence of hypopituitarism Outpatient follow-up duration (months) Type of clinic (specialized pituitary clinic)	+0.19 +0.02 -0.2 -0.025 +0.26	4.1−32.8 −13.4−17 −39.1 to −3.38 −0.3−0.2 9.5−38.17	0.01* 0.8 0.02* 0.74 0.001*
**Role Emotional** *Model summary*: R² = 0.1; ANOVA *p* < 0.001* ** Predictor**	**β**	**95% CI**	**p-value**
Sex (female) History of pituitary surgery Presence of hypopituitarism Outpatient follow-up duration (months) Type of clinic (specialized pituitary clinic)	+0.19 -0.06 -0.17 -0.09 +0.28	4.4−33.5 −21.4−9.5 −37 to −0.9 −0.4−0.1 10.9−39.9	0.01* 0.4 0.03* 0.2 0.001*
**Social Functioning** *Model summary*: R² = 0.1; ANOVA *p* < 0.001* ** Predictor**	**β**	**95% CI**	**p-value**
Sex (female) History of pituitary surgery Presence of hypopituitarism Outpatient follow-up duration (months) Type of clinic (specialized pituitary clinic)	+0.23 -0.01 -0.19 -0.12 +0.2	5.7−24.7 −11.1−9 −25.5 to −1.7 −0.2−0.1 2.6−21.5	0.002* 0.8 0.02* 0.9 0.01
**Bodily Pain** *Model summary*: R² = 0.14; ANOVA *p* < 0.001* ** Predictor**	**β**	**95% CI**	**p-value**
Sex (female) History of pituitary surgery Presence of hypopituitarism Outpatient follow-up duration (months) Type of clinic (specialized pituitary clinic)	+0.3 +0.08 -0.12 -0.07 +0.2	9.8−28 −5.1−14.2 −19.9−2.7 −0.2−0.09 2.5−20.6	< 0.001* 0.3 0.1 0.3 0.01*

* *p* < 0.05 was considered statistically significant. All models were adjusted for sex, history of pituitary surgery, presence of hypopituitarism, outpatient follow-up duration, and clinic type. Abbreviations: β, standardized regression coefficient; CI, confidence interval

### Comparison of illness perception subscale scores between centers

In the comparison between centers, significant differences were observed across several Illness Perception Scale subscales. Patients followed in the specialized pituitary clinic exhibited higher scores in the Timeline (acute/chronic), Consequences, and Treatment Control subscales, whereas patients followed in the general endocrinology clinics demonstrated higher scores in the Risk Factors and Chance subscales (*p* = 0.001, *p* < 0.001, *p* < 0.001, *p* < 0.001, and *p* = 0.036, respectively) (Table 2).

To determine whether between-group differences in sex, history of pituitary surgery, hypopituitarism, duration of follow-up, or clinic type accounted for these findings, multivariable regression analyses were performed. In the adjusted models, Timeline scores were independently associated with longer duration of outpatient follow-up (*p* = 0.02) and follow-up in the specialized pituitary clinic (*p* = 0.002). Consequences scores were higher among patients with a history of pituitary surgery (*p* = 0.003) and those followed in the specialized clinic (*p* = 0.05). Treatment Control scores were independently associated with shorter duration of outpatient follow-up (*p* = 0.03) and follow-up in the specialized pituitary clinic (*p* = 0.02). Risk Factors scores were higher among patients managed in general endocrinology clinics (*p* = 0.001). For the Chance subscale, the regression model demonstrated low explanatory power (R² = 0.02), and none of the demographic or clinical variables showed a statistically significant association. Detailed multivariable regression results are presented in Table 4.

**Table 4:** Multivariable regression analyses of factors associated with illness perception subscale scores

**Timeline (acute/chronic)** *Model summary*: R² = 0.13; ANOVA *p* < 0.001* **Predictor**	β	95% CI	*p*-value
Sex (female) History of pituitary surgery Presence of hypopituitarism Outpatient follow-up duration (months) Type of clinic (specialized pituitary clinic)	-0.06 -0.12 +0.12 +0.17 -0.24	−0.5−0.2 −0.7−0.1 −0.13−0.8 0−0.17 −0.9 to −0.21	0.3 0.1 0.1 0.02* 0.002*
**Consequences** *Model summary*: R² = 0.14; ANOVA *p* < 0.001* ** Predictor**	**β**	**95% CI**	**p-value**
Sex (female) History of pituitary surgery Presence of hypopituitarism Outpatient follow-up duration (months) Type of clinic (specialized pituitary clinic)	-0.14 -0.25 +0.08 -0.03 -0.15	−0.8−0.1 −1.1 to −0.2 −0.2−0.8 −0.0−0.0 −0.8−0.1	0.06 0.003* 0.3 0.9 0.05*
**Treatment Control** *Model summary*: R² = 0.09; ANOVA *p* = 0.006* ** Predictor**	**β**	**95% CI**	**p-value**
Sex (female) History of pituitary surgery Presence of hypopituitarism Outpatient follow-up duration (months) Type of clinic (specialized pituitary clinic)	+0.01 +0.01 +0.06 -0.16 -0.18	−0.17−0.2 −0.18−0.2 −0.14−0.3 −0.008 to −0.00 −0.4 to −0.29	0.8 0.8 0.4 0.03* 0.02*
**Risk Factors** *Model summary*: R² = 0.12; ANOVA *p* < 0.001* ** Predictor**	**β**	**95% CI**	**p-value**
Sex (female) History of pituitary surgery Presence of hypopituitarism Outpatient follow-up duration (months) Type of clinic (general endocrinology clinic)	+0.13 +0.0 -0.12 +0.07 +0.28	−0.02−0.3 −0.19−0.2 −0.4−0.05 −0.002−0.006 0.15−0.5	0.07 0.9 0.1 0.3 0.001*

* *p* < 0.05 was considered statistically significant. All models were adjusted for sex, history of pituitary surgery, presence of hypopituitarism, outpatient follow-up duration, and clinic type. Abbreviations: β, standardized regression coefficient; CI, confidence interval

### Comparison of outpatient satisfaction subscale scores between centers

In the comparison between centers, all subscales of the Outpatient Satisfaction Scale, including Appointment Procedures, Effective Clinical Encounter, Staff Attitude, Waiting Time and Counseling, and Overall Satisfaction, as well as the total scale score differed significantly between groups. Patients followed in the specialized pituitary clinic demonstrated higher scores across all subscales (*p* < 0.001, *p* < 0.001, *p* = 0.002, *p* = 0.006, *p* < 0.001, and *p* < 0.001, respectively) (Table 2).

To determine whether between-group differences in sex, history of pituitary surgery, hypopituitarism, duration of outpatient follow-up, or clinic type accounted for these findings, multivariable regression analyses were performed. In the adjusted models, higher Appointment Procedures scores were independently associated with prior pituitary surgery (*p* = 0.001) and follow-up in the specialized pituitary clinic (*p* < 0.001). Effective Clinical Encounter scores were predicted by shorter outpatient follow-up duration and specialized clinic follow-up (both *p* < 0.01). The regression model for Staff Attitude demonstrated low explanatory power (R² = 0.03), and none of the examined variables showed a statistically significant association. Higher Waiting Time and Counseling scores were independently associated with shorter outpatient follow-up duration (*p* = 0.005). Higher Overall Satisfaction scores were associated with shorter follow-up duration and follow-up in the specialized clinic (*p* = 0.002, and *p* < 0.001, respectively). Similarly, higher total Outpatient Satisfaction Scale scores were predicted by shorter follow-up duration and specialized clinic follow-up (*p* = 0.008, and *p* < 0.001, respectively) (Table 5).

**Table 5:** Multivariable regression analyses of factors associated with outpatient satisfaction subscale scores

Appointment Procedures *Model summary*: R² = 0.2; ANOVA *p* < 0.001* Predictor	β	95% CI	*p*-value
Sex (female) History of pituitary surgery Presence of hypopituitarism Outpatient follow-up duration (months) Type of clinic (specialized pituitary clinic)	-0.05 -0.27 -0.05 -0.07 -0.23	−0.4−0.2 −1 to −0.25 −0.6−0.29 −0.01−0.004 −1 to −0.3	0.4 0.001* 0.4 0.3 < 0.001*
**Effective Clinical Encounter** *Model summary*: R² = 0.28; ANOVA *p* < 0.001* ** Predictor**	**β**	**95% CI**	**p-value**
Sex (female) History of pituitary surgery Presence of hypopituitarism Outpatient follow-up duration (months) Type of clinic (specialized pituitary clinic)	+0.01 -0.04 +0.06 -0.16 -0.42	−0.14−0.19 −0.22−0.12 −0.11−0.3 −0.08 to −0.001 −0.6 to −0.32	0.8 0.5 0.3 0.01* < 0.001*
**Waiting Time and Counseling** *Model summary*: R² = 0.08; ANOVA *p* = 0.013* ** Predictor**	**β**	**95% CI**	**p-value**
Sex (female) History of pituitary surgery Presence of hypopituitarism Outpatient follow-up duration (months) Type of clinic (specialized pituitary clinic)	+0.05 -0.01 -0.06 -0.22 -0.08	−0.16−0.37 −0.32−0.25 −0.4−0.21 −0.01 to −0.002 −0.41−0.12	0.4 0.8 0.4 0.005* 0.2
**Overall Satisfaction** *Model summary*: R² = 0.3; ANOVA *p* < 0.001* ** Predictor**	**β**	**95% CI**	**p-value**
Sex (female) History of pituitary surgery Presence of hypopituitarism Outpatient follow-up duration (months) Type of clinic (specialized pituitary clinic)	-0.02 +0.02 +0.05 -0.21 -0.47	−0.28−0.18 −0.2−0.29 −0.18−0.4 −0.012 to −0.003 −1 to −0.5	0.6 0.7 0.4 0.002* < 0.001*
**Total Outpatient Satisfaction Scale Score** *Model summary*: R² = 0.3; ANOVA *p* < 0.001* ** Predictor**	**β**	**95% CI**	**p-value**
Sex (female) History of pituitary surgery Presence of hypopituitarism Outpatient follow-up duration (months) Type of clinic (specialized pituitary clinic)	-0.17 -0.11 +0.006 -0.18 -0.4	−0.19−0.153 −0.32−0.04 −0.2−0.22 −0.08 to −0.001 −0.67 to −0.32	0.8 0.1 0.9 0.008* < 0.001*

* *p* < 0.05 was considered statistically significant. All models were adjusted for sex, history of pituitary surgery, presence of hypopituitarism, outpatient follow-up duration, and clinic type. Abbreviations: β, standardized regression coefficient; CI, confidence interval

Additionally, among patients followed in the specialized pituitary clinic, responses to the item evaluating the perceived contribution of support staff showed that 77.3% selected ‘Strongly Agree,’ 18.6% selected ‘Agree,’ and 2.1% selected either ‘Undecided’ or ‘Disagree.’

In the overall cohort, patients with comorbidities demonstrated lower SF-36 Physical Functioning, Role Physical, Role Emotional, Social Functioning, and General Health Perception scores (all *p* < 0.05). In center-specific analyses, no significant differences were observed within the specialized pituitary clinic, whereas in general endocrinology clinics, comorbidity was associated with lower Physical Functioning, Role Physical, and General Health Perception scores (all *p* < 0.05). For the Illness Perception Scale, overall analyses showed higher Consequences scores in patients with comorbidities (*p* = 0.007). In general endocrinology clinics, comorbidity was additionally associated with higher Symptom Identity, Consequences, Psychological Attributions, Risk Factors, and Immunity scores (all *p* < 0.05), while no significant differences were observed in the specialized pituitary clinic (all *p* > 0.05). Outpatient Satisfaction scores were comparable (all *p* > 0.05); however, Waiting Time and Counseling score was higher among patients with comorbidities in general endocrinology clinics (*p* = 0.01).

In exploratory analyses comparing functioning and non-functioning adenomas, no significant differences were observed across SF-36 domains, Illness Perception Scale subscales, or Outpatient Satisfaction Subscale scores in the overall cohort or within the specialized pituitary clinic group (all *p* > 0.05). In the general endocrinology clinic group, patients with functioning adenomas showed higher Role Physical scores compared with those with non-functioning adenomas (*p* = 0.018).

Within the acromegaly subgroup, patients receiving injectable therapy and those not receiving injections were comparable in terms of sex, educational level, pituitary surgery, radiotherapy, hypopituitarism prevalence, and IGF-1 levels (all *p* > 0.05), whereas GH levels were higher in the injectable therapy group (1.34 [0.7–4.6] µg/L vs. 0.6 [0.3–1.9] µg/L; *p* = 0.044). Across the acromegaly subgroup, SF-36 domains and Outpatient Satisfaction Subscale scores were comparable between patients receiving and not receiving injectable therapy (all *p* > 0.05) and were also similar across centers (all *p* > 0.05). Within the Illness Perception Scale, only the Chance subscale differed (*p* = 0.03), with higher scores observed in patients not receiving injectable treatment.

## Discussion

In this study, we observed that patient-reported outcomes differed meaningfully between a PTCOE-aligned specialized pituitary care and general endocrinology settings, suggesting that the organization of pituitary care may be associated with differences in how patients experience and interpret their illness. Based on SF-36 domains, patients followed in the specialized clinic reported patterns reflecting greater functional limitations, emotional strain, reduced social engagement, and more prominent pain-related interference, consistent with a population managing a heavier clinical and psychosocial burden. In parallel, illness-perception profiles derived from the IPS indicated that these patients were more likely to view their condition as chronic, consequential, and responsive to treatment, suggesting heightened disease awareness and stronger perceived treatment controllability. By contrast, patients followed in general endocrinology clinics more frequently attributed their illness to diffuse or external causes, including chance, a pattern associated with lower perceived control. Despite these differences in functional status and illness perceptions, satisfaction with outpatient care was consistently higher among patients treated in the specialized clinic. Notably, clinic type independently contributed to variation across several PROs subscales, suggesting that differences in care organization may have influenced patient experience beyond clinical factors alone. Importantly, these differences should be interpreted as reflecting underlying disease-related complexity rather than causal effects of the care setting itself. These findings were further supported by multivariable analyses, which indicated that observed patterns were shaped not only by clinic structure but also by key patient-level characteristics, including sex, presence of hypopituitarism, surgical history, and duration of follow-up, highlighting the interplay between clinical complexity and care organization.

The SF-36 is a robust and widely validated instrument for assessing health-related quality of life, encompassing both physical and psychosocial dimensions of well-being, including limitations in daily functioning, emotional distress, social participation, and the overall impact of symptoms on everyday life [29]. In pituitary disorders, the SF-36 has been consistently utilized to delineate global disease burden and long-term functional consequences across diverse pituitary disease populations [14, 35–37]. In our study, patients followed in the specialized pituitary clinic demonstrated significantly lower scores in the Role Physical, Role Emotional, Social Functioning, and Bodily Pain domains of the SF-36, indicating a greater perceived functional and psychosocial burden. Declines in these domains reflect concrete challenges in daily life: lower Role Physical scores indicate greater difficulty performing routine activities; reduced Role Emotional scores correspond to diminished emotional resilience; decreased Social Functioning scores denote restricted social engagement and impaired interpersonal participation; and lower Bodily Pain scores reflect heightened pain interference that can further limit mobility, activity tolerance, and overall quality of life. Collectively, these findings reflect a multidimensional functional and psychosocial burden [29]. This pattern aligns with existing literature showing that individuals with chronic pituitary disorders, particularly acromegaly, Cushing’s disease, and postoperative hypopituitarism, often experience persistent impairments in physical and emotional well-being despite biochemical control [6, 38, 39].

In exploratory subgroup analyses, functioning adenomas within the general endocrinology clinic demonstrated higher Role Physical scores than non-functioning adenomas, possibly reflecting differences in mass effect or hypopituitarism burden. Specialized centers typically serve more complex referrals, including patients with longer disease duration, prior surgical interventions, and higher hypopituitarism prevalence, factors that have been independently associated with reduced quality-of-life outcomes in multiple studies [3, 6, 40]. Our multivariable analysis supports this interpretation, as both hypopituitarism and surgical history emerged as significant predictors of lower domain scores. Female sex similarly emerged as an independent predictor, consistent with prior evidence showing that female sex adversely influences SF-36 outcomes in chronic endocrine conditions [41, 42]. This association may reflect sex-related differences in symptom perception, fatigue sensitivity, and psychosocial vulnerability described in chronic endocrine conditions [41]. Critically, the specialized clinic managed a markedly more complex patient population, with significantly higher rates of prior pituitary surgery (63.9% vs. 32.1%) and hypopituitarism (28.9% vs. 14.1%), both well-established determinants of reduced quality of life in pituitary disease. Interpreting these lower SF-36 scores without accounting for this differential disease burden would therefore be misleading, as the disproportionate representation of surgically treated and hypopituitary patients in the specialized clinic likely accounts for a substantial portion of the observed between-group differences. Overall, the lower quality-of-life scores observed in the specialized clinic appear to reflect a greater underlying disease burden rather than limitations of the care model itself. This pattern likely reflects the referral structure of the specialized clinic, which retains surgically treated and clinically complex patients for long-term multidisciplinary follow-up rather than discharging them to general endocrinology care, a case-mix effect that should be considered when interpreting the SF-36 comparisons. These observations are consistent with health-services research highlighting the role of care coordination, continuity, and perceived support in shaping patient-reported outcomes [43]. Accordingly, integrating structured psychosocial assessment, fatigue management, and targeted endocrine rehabilitation into specialized pituitary pathways may better address multidimensional patient needs.

The Illness Perception Scale is a theory-based instrument that assesses patients’ cognitive and emotional representations of their illness, including beliefs related to chronicity, consequences, causation, and perceived control [31, 32]. Unlike generic quality-of-life measures, the IPS does not quantify functional impairment per se but provides insight into how patients interpret, make sense of, and emotionally respond to their disease [31, 32]. These perceptions are clinically relevant, as maladaptive illness beliefs have been associated with poorer treatment adherence and adverse psychosocial outcomes, whereas greater perceived treatment control and illness coherence may support engagement and coping, even in the context of chronic disease [31, 32]. In line with this conceptual framework, the scale has been applied across a broad spectrum of conditions, ranging from relatively minor disorders such as allergic conditions and headache disorders to serious and chronic diseases including cancer and diabetes, reflecting its versatility in capturing how individuals interpret and adapt to illness [44, 45]. Previous studies have also employed the IPS in pituitary disorders, supporting its relevance for chronic endocrine disease populations [46]. In our cohort, patients followed in the specialized pituitary clinic exhibited higher Timeline, Consequences, and Treatment Control scores, indicating stronger perceptions of chronicity, disease impact, and treatment engagement. Prior studies similarly report that patients with long-standing or surgically treated pituitary disease often perceive their illness as more persistent and burdensome [46, 47]. The association between higher Consequences scores and surgical history in our study supports this interpretation. Conversely, patients followed in general endocrinology clinics demonstrated higher Risk Factors and Chance attributions, suggesting more externalized illness models. In the acromegaly subgroup, Chance attribution scores were higher among patients not receiving injectable therapy. Prior treatment-specific studies indicate that even biochemically controlled patients receiving somatostatin receptor ligands may report persistent symptom burden [48], and treatment satisfaction correlates with psychological status in acromegaly [49]. Thus, higher Chance attribution may reflect differences in illness appraisal rather than objective severity. These attribution styles may influence psychological well-being, self-management, and adherence in chronic endocrine disease [50]. Comorbidity burden similarly contributed to less favorable patient-reported outcomes across both quality-of-life and illness perception measures, further supporting the role of overall health status in shaping perceived disease impact. Understanding how care environments shape illness perceptions is therefore essential for tailoring education and sustaining long-term engagement. One potential mechanism relates to the structure and intensity of patient support embedded within specialized pituitary clinics. In our cohort, the vast majority of patients followed in the specialized clinic reported that the presence of support staff contributed positively to the quality of their clinical follow-up, suggesting that enhanced staff accessibility may facilitate clearer communication and reinforce perceived treatment control. Although causality cannot be inferred, structured nurse involvement, adequate consultation time, and access to psychosocial support appear to represent modifiable components for optimizing patient engagement.

Patient satisfaction is a key patient-reported outcome reflecting patients’ experiences with healthcare delivery beyond clinical outcomes, encompassing access, communication, staff attitude, and coordination [18, 34]. The Outpatient Satisfaction Scale and related patient experience instruments have been widely applied in patients with hypothalamic–pituitary and other endocrine disorders [18, 34, 51]. In our study, satisfaction scores were consistently higher in the specialized pituitary clinic, supporting an association between multidisciplinary PTCOE-aligned care and more coordinated service delivery. This finding aligns with prior evidence indicating that integrated endocrine–neurosurgical pathways, streamlined appointment processes, and enhanced access to disease-specific expertise contribute to improved care quality and patient engagement [8]. Importantly, outpatient satisfaction scores observed in general endocrinology clinics were not poor, with mean subscale scores remaining above nationally reported averages, suggesting an overall acceptable level of service quality within these settings [34]. This likely reflects the subspecialty-oriented nature of general endocrinology practice within tertiary centers. However, the consistently higher satisfaction scores observed in the specialized pituitary clinic suggest that increasing degrees of disease-specific specialization, particularly within structured, multidisciplinary pituitary care models, may confer incremental benefits beyond standard subspecialty care, rather than reflecting a simple dichotomy between ‘specialized’ and ‘non-specialized’ services [8]. In multivariable models, follow-up in the specialized clinic and shorter outpatient follow-up duration emerged as the strongest predictors of higher satisfaction scores, highlighting the importance of communication clarity and structured counseling. Overall, these findings reveal a clinically meaningful dissociation: despite managing a population with substantially greater disease burden and lower quality-of-life scores, the PTCOE-aligned clinic achieved superior satisfaction outcomes across all subscales. This pattern underscores that organizational structure, multidisciplinary coordination, and disease-specific expertise appear to be associated with a distinctly positive patient experience, independent of the patient’s underlying clinical complexity, and represents one of the key messages of this study.

This study has several notable strengths. To our knowledge, it represents one of the first comparative assessment in the existing literature examining patient-reported outcomes across a PTCOE-aligned specialized pituitary clinic and general endocrinology clinics, thereby providing a comparative framework for evaluating how care organization may be associated with patient-reported outcomes. The use of three validated and culturally adapted patient-reported outcome instruments allowed for a comprehensive, multidimensional assessment of patient experience, while the inclusion of two high-volume tertiary centers enhances the real-world applicability of the findings. Multivariable analytical methods strengthened the interpretability of observed associations by accounting for key clinical covariates. Nonetheless, several limitations should be acknowledged. The cross-sectional design restricts causal inference, and residual confounding from unmeasured psychosocial characteristics cannot be fully excluded. In addition, disease-specific patient-reported outcome instruments, such as AcroQoL for acromegaly or CushingQoL for Cushing’s disease, were not employed, which may have limited the sensitivity of the assessment to capture condition-specific aspects of patient experience. The use of generic instruments was nonetheless deliberate: given the heterogeneous diagnostic composition of the cohort, encompassing four distinct pituitary adenoma subtypes, generic measures such as the SF-36 facilitate cross-diagnostic comparisons and enable benchmarking against normative population data, strengths that are more difficult to achieve with disease-specific tools in a mixed-diagnosis cohort setting. Differences in follow-up duration partly reflect institutional chronology, as the specialized clinic was established in 2020 whereas the general endocrinology clinic has operated since 2002, resulting in systematically longer follow-up in the latter setting. Furthermore, the two centers serve patient populations through distinct referral pathways, with the specialized clinic selectively managing more complex and surgically treated cases. This referral-related selection bias may have contributed to the observed between-group differences and cannot be fully accounted for in the current analyses. Additionally, although the specialized clinic aligns with key PTCOE principles, the absence of formal international PTCOE certification may limit direct comparability with fully accredited pituitary tumor centers and should be considered when interpreting the generalizability of the findings. Finally, the study was conducted in a single metropolitan region, which may limit broader generalizability across diverse healthcare systems or PTCOE models.

In conclusion, this study highlights that specialized pituitary clinics structured within a PTCOE-aligned, though not formally accredited, model was associated with a distinct patient experience characterized by clearer care pathways, more coherent illness representations, and higher satisfaction, despite serving a clinically more complex population. These findings support incorporating PROs into pituitary service evaluation and suggest that organizational design and care coordination appear to be associated with patient engagement and care experience. Future longitudinal and multi-center studies are warranted to assess how specialized care models shape long-term functional outcomes and to further refine patient-centered performance metrics within PTCOE frameworks. Given that the specialized clinic has not undergone formal international PTCOE accreditation, generalizability to fully certified PTCOEs warrants caution and replication in accredited centers is encouraged.

## Data Availability

The data underlying this article are available from the corresponding author upon reasonable request.
